# Immoral Publications

**Published:** 1868-11

**Authors:** Henry Gibbons


### Immoral Publications.

"I cannot dwell with too much emphasis on the important practical distinction between the moral treatment which inspires confidence and hope, and tends to dispel the cloud of anxiety and apprehension, and that which confirms suspicion and excites alarm; between that which is prompted by professional and honorable motives, and which has the good of the patient for its primary object, and that which is controlled by the love of gain, and makes body and soul the sport of the vilest tricks of trade. The system of empirical advertising may be regarded as an ingenious device for robbing men of their money, their happiness, and their reason. The newspaper, going into every house, carries a flood of this poisonous literature. The newspaper makes our females familiar with the idea of the prevention of pregnancy, demoralizing the married and corrupting the unmarried. The newspaper prompts the idea of abortion, and indirectly encourages a criminal practice so wide-spread as to disturb the foundations of society, and to affect the national welfare. The newspaper furnishes the abortionist with the means of inviting all the mothers in the land to murder their unborn offspring through his nefarious agency. The newspaper is a daily reminder to every villain who plots the ruin of females, how he can accomplish his diabolical purpose without exposure. The newspaper puts in the hands of every boy and every girl, in city and country, a daily stream of impure, obscene and corrupting literature, which they could find nowhere else.

"The reader will not understand me as pronouncing a censure on the conductors of the press in this relation. I remember too well the example of the knight and the windmill. The laws of trade govern the press, and mould the code of morals for this, in common with other departments of industry. I will do the proprietors of newspapers the justice to declare my conviction that they all believe and know that the advertisements referred to are unfit for general reading, and that they are morally pernicious. I believe further, that scarcely one newspaper on this coast would admit such advertisements, if others did not.

"The same defense applies to those respectable druggists who suffer their names to be announced as venders and endorsers of universal cures for nervous weakness and impotence, and female obstructions. It is well understood that people of intelligence will not believe the statements which are made, and that only credulous and weak-minded persons, composing not much over nine-tenths of the community, will be misled and fleeced by such publications.

"If writings published with the design of doing good are capable of so much evil, as appears from what I have previously said, how much more mischief is calculated to result from publications artfully prepared for the very purpose of frightening the reader into the belief that he is sick, and which are daily thrust before his eyes, so that he cannot avoid seeing them if he would! I have an earnest conviction that the literature of quackery is much more pernicious than is commonly supposed—that few persons suspect the depth to which it strikes its roots into the corporeal, moral, and intellectual life of society, and the consequent amount of vice, trouble, disease and insanity which it produces.—Dr. Henry Gibbons, *Editor Pacific Medical and Surgical Journal.*

